# Time–Frequency–Energy Characteristics Analysis of Vibration Signals in Digital Electronic Detonators and Nonel Detonators Exploders Based on the HHT Method

**DOI:** 10.3390/s23125477

**Published:** 2023-06-10

**Authors:** Haojie Yin, Hui Chen, Yin Feng, Jingkun Zhao

**Affiliations:** 1 School of Geology and Mining Engineering, Xinjiang University, Urumqi 830046, China; yinhaojie@stu.xju.edu.cn (H.Y.);; 2 Key Laboratory of Environmental Protection Mining for Minerals Resources, Education Department of Xinjiang Uygur Autonomous Region, Xinjiang University, Urumqi 830046, China; 3 School of Resources and Safety Engineering, Central South University, Changsha 410083, China

**Keywords:** rock roadway excavation and blasting, digital electronic detonators, nonel detonators, Hilbert–Huang Transform method, signal analysis, particle size analysis

## Abstract

The China Society of Explosives and Blasting required a larger than 20% annual increase in the national use of digital electronic detonators since 2018. So, this article conducted a large number of on-site tests and then used the Hilbert–Huang Transform method to analyze and compare the vibration signals of digital electronic and nonel detonators during the excavation process of minor cross-sectional rock roadways from the perspective of time, frequency, and energy. Then, through vibration energy analysis, identification of actual delay time, and formula derivation, it was proved that the delay time error of the detonator can control vibration wave random interference and reduce vibration. The analysis results showed that when using a segmented simultaneous blasting network for excavation in small-sectioned rock tunnels, nonel detonators may provide more excellent protection to structures than digital electronic detonators. In the same segment, the timing error of nonel detonators produces a vibration wave with a random superposition damping effect, resulting in an average vibration reduction of 19.4% per segment compared to digital electronic detonators. However, digital electronic detonators are superior to nonel detonators for the fragmentation effect on rock. The research conducted in this paper has the potential to facilitate a more rational and comprehensive promotion of digital electronic detonators in China.

## 1. Introduction

China’s civil explosive industry is rapidly moving towards refined blasting, and the continuous innovation of digital electronic detonator technology has taken the initiative to a new level [1,2,3,4]. The civil explosive industry widely promotes digital electronic detonators for their advantages, such as accurate delay timing and enhanced safety. Integrated circuit chips allow for the arbitrary selection of delay time within 1-16 s, achieving exceptionally high accuracy with a delay error of only about 0.1 ms [5,6]. According to multiple on-site surveys, most of the Xinjiang mines are transitioning from using the nonel detonator to a digital electronic detonator. The National Ministry of Industry and Information Technology’s safety production department and the public security bureau’s security management department require the full domestic implementation of digital electronic detonators by the end of 2022. Against this backdrop, exploring the advantages and disadvantages of the two detonators in terms of vibration signal time–frequency–energy and crushing effect has important guiding significance for on-site construction.

Blasting operations widely employ digital electronic and nonel detonators, and drilling and blasting methods serve as essential excavation techniques in mining, quarrying, and civil engineering projects such as tunnel excavation. [7,8]. However, using explosives and detonators brings various safety concerns, including blast-induced vibrations, flying rocks, impact, and noise [9,10,11]. Among these safety issues, the hazards associated with blast-induced vibrations are of particular concern [12,13,14], as the energy contained in the vibrations can not only cause damage to structures but also disrupt the lives of residents [15,16]. Therefore, it is crucial to accurately measure on-site vibrations and analyze blast-induced vibrations’ intrinsic temporal, spectral, and energy characteristics [17]. Currently, common methods for analyzing blast-induced vibration signals include wavelet packet transform [18,19,20], wavelet analysis [21,22], Fourier transform [23,24,25], and Hilbert–Huang transform (HHT) [26,27]. However, each of these methods has its limitations in analyzing vibration signals. Fourier transform has significant limitations in analyzing highly nonlinear and non-stationary vibration signals with rapid changes. Although wavelet and wavelet packet analyses theoretically can analyze nonlinear and non-stationary vibration signals, they do not fully resolve the issue of analyzing unstable information in practical algorithms. In contrast, HHT, through empirical mode decomposition (EMD) and Hilbert transform, truly achieves analysis of nonlinear and non-stationary signals, overcoming the challenges faced by other signal analysis methods. The high adaptability of HHT allows it to generate adaptive bases, distinguishing it from the limitations of Fourier transform and wavelet transform, which are restricted to a fixed set of predetermined bases. The analysis by HHT provides instantaneous frequency, which exhibits local features. The accuracy of the frequency obtained through wavelet analysis and Fourier transform is far inferior to that obtained through HHT analysis. Therefore, HHT was chosen as the primary research method for this vibration wave analysis.

Specifically, the comparative study between digital electronic and nonel detonators mainly includes the effect of rock fragmentation or the spatial shaping effect during underground blasting excavation and the comparison of blasting vibration effects [28,29]. However, ultimately, the reason for the differences in the application of these two types of detonators on site lies in the accuracy and adjustable range of the delay time of the detonators. Digital electronic detonators are currently widely promoted due to their reliable control of the delay time. However, their actual cost is roughly ten times that of traditional detonating cords, significantly hindering their comprehensive promotion [30,31]. Fortunately, the impact of cost is not absolute. For example, in open-pit mining with terraced and tunnel blasting, using detonating cords for staged simultaneous blasting is not a suitable choice due to many blast holes, both in terms of vibration effects and fragmentation effects. However, this situation is ideal for using digital electronic detonators for sequential blasting, exploring the best delay time for overlapping vibration damping based on the theory of blasting vibration superposition, which can significantly reduce the harm of blasting vibration [32]. On the other hand, numerous field tests have also shown that sequential blasting with digital electronic detonators results in a lower large block rate of fractured rock, greatly improving the efficiency of excavation, loading, and transportation while somewhat reducing later-stage costs, which balances their cost to some extent [33].

However, due to the small tunnel section and few boreholes, contractors generally use segmented simultaneous detonation blasting networks for small cross-section rock tunnel excavation and blasting. Precisely controlling the delay time between segments and achieving simultaneous detonation of boreholes within each segment positively affects the blasting effect. However, through many field experiments, it has been found that under the same conditions, the vibration caused by nonel detonators is smaller than that caused by digital electronic detonators. Moreover, when comparing the two types of detonators, most scholars often focus on the large-scale blasting of open-pit mine benches or the excavation and blasting of large cross-section tunnels. In such working conditions, there is no doubt that digital electronic detonators are far superior to nonel detonators in reducing blasting vibration hazards or controlling the blasting effect. However, there is relatively little comparative research on segmented blasting of small cross-section rock tunnel excavation. Therefore, in this paper, the HHT method was used to compare and analyze the time-frequency energy characteristics of the blasting vibration signals between digital electronic detonator initiation and nonel detonator initiation based on the measured data of blasting vibration in a small cross-section rock tunnel of a mine. The envelope spectra of the vibration signals of the two types of detonators were obtained through EMD. The delay time errors of the detonators were accurately identified, and it is determined that the simultaneous blasting rule is not followed among the boreholes within the same segment for nonel detonators.

Furthermore, the conditions for the disorderly superposition of vibration waves caused by the delay error between boreholes within the same segment of nonel detonators, resulting in a vibration reduction, are explored through formula derivation. Finally, the blasting fragmentation analysis software Wipware compares and analyzes the blasting effect of the two types of detonators under the same working conditions. The analysis results provide a theoretical basis for reducing costs and improving efficiency, controlling blasting vibration, and further promoting the comprehensive application of digital electronic detonators.

The main innovations of this paper are as follows:

- This study focused on the frequently overlooked excavation and blasting of small-section rock tunnels, presenting new experimental findings that comprehensively compare digital electronic detonators and nonel detonators;
- The study uncovered the capability of nonel detonators to reduce vibration wave interference by actively controlling delay time errors;
- The study effectively found the range of delay time errors for nonel detonators to mitigate vibration wave interference through random stacking.

## 2. Experiment Method

### 2.1. Field Test

The Bei-Zhan iron ore mining area is located at a distance of 160 km in the direction of 327° from Hejing County, with a direct distance of 84 km from Baluntai in Hejing County and a road distance of 130 km.

**Figure 1:**
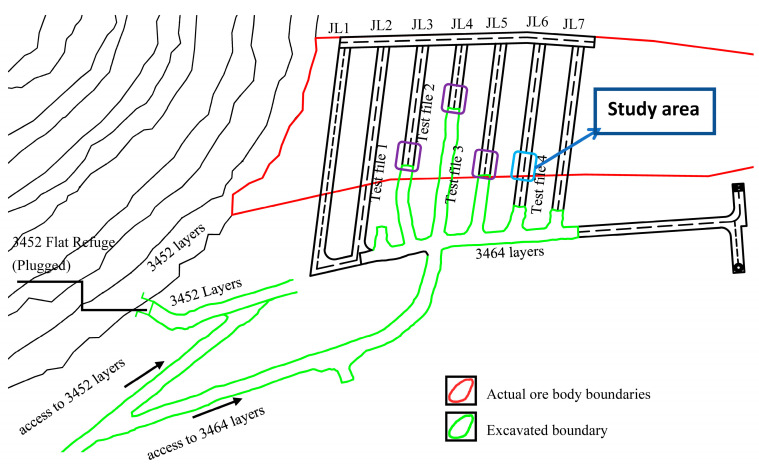
Schematic diagram of the location of the test mining area.

Its administrative jurisdiction belongs to Bayinguoleng Mongolian Autonomous Prefecture Hejing County. Since 27 September 2021, multiple vibration tests have been carried out in the mine. In this test, the No. 4 ore removal tunnel was selected, as shown in Figure 1, and vibration tests and analysis of fragmentation degree were conducted on the digital electronic detonators and nonel detonators during the excavation and blasting. It should be noted that the primary working conditions of the two different sets of detonators are nearly identical, including the target rock, the segmented blasting stages, the hole network parameters, the total amount of explosives used, the explosives used per borehole, the type of explosives, and the layout of monitoring points, as indicated in Figure 1. The purple boxed area in Figure 1 represents the current excavation locations of other tunnels, while the blue boxed area represents the test site for this experiment.

#### 2.1.1. Blasting Construction Plan

**Figure 2:**
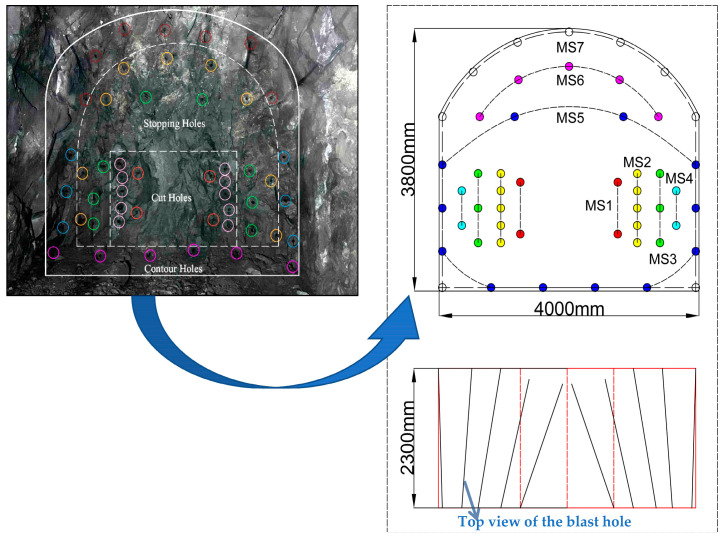
Blasting hole layout plan and top view.

A double-wedge-shaped slotting method was used for the working face during the testing period. The measured average hole depth of the first round of slotting was 1.14 m, and that of the second round was 2.07 m. The auxiliary and peripheral holes were 2.2–2.3 m in depth, and the diameter of the blasting hole was 40 mm. The explosive used for this blasting was a bar-shaped emulsion explosive with dimension parameter Φ32 mm × 320 mm × 300 g (diameter × length × mass). The delay interval between digital electronic detonators is set at 150 ms for 7-segment blasting. We selected 1/2 s delay nonel detonators, and the selected segments were classified into the 1st, 2nd, 3rd, 4th, 5th, 6th, and 9th segments for segmented blasting, with simultaneous blasting of boreholes within the same segment. Fifty blasting holes were fired in each cycle (including 4 for the first round of slotting, 10 for the second round, 17 for the auxiliary holes, and 19 for the peripheral holes), consuming a total explosive mass of 66 kg. The maximum explosive consumption for a single blast segment is 16.2 kg, and the advance per cycle is about 2.1 m. The number of roll-packed emulsion explosives used for each section (MS1–MS7) was 12, 50, 30, 20, 54, 20, and 34, respectively. The blasting hole connection is shown in Figure 2, and the blasting sequence was conducted from left to right and from top to bottom, according to the legend in the figure. In Figure 2, solid circles of the same color represent boreholes detonated at the same time point.

#### 2.1.2. Monitoring Point Layout Plan

The testing in this study utilized the iSensor series three-axis intelligent sensors produced by Topu Company in Sichuan Province, China. The accuracy of this sensor meets national Class A standards, with a resolution of 16 bits and high data acquisition accuracy, which did not affect the analysis results. The instrument collection parameters were set to continuous collection mode, negative delay length of 256 ms, collection duration of 10 s, and the maximum number of segments that can be recorded are 65,535. The channel range was set to 40 g, and the instrument had a fixed built-in sampling rate of 4000 Hz. A total of 6 vibration meters were used, and the same collection parameters were set after the offline collection, connected to the terminal device to download the data.

**Figure 3:**
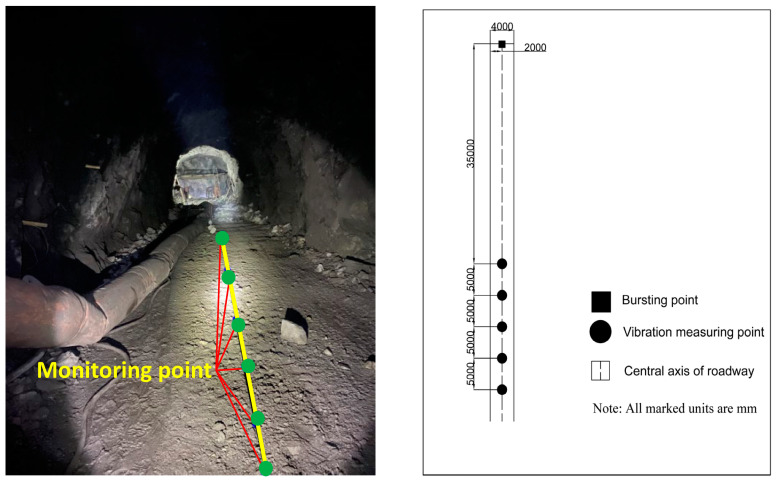
Layout schematic of monitoring points for No.3464-4 entry: (**a**) Experimental point photos and monitoring point annotations; (**b**) Layout CAD schematic diagram of monitoring points.

As shown in Figure 3, this study used a straight-hole layout method along the centerline of the roadway floor. The first monitoring point was 35 m from the face, and the distance between adjacent sensor intervals was 5 m. The number of instruments to be installed was determined according to the conditions of the test site, with no less than three instruments and no more than six instruments used for each monitoring. The *X*-axis of the sensor was aimed at the blast center.

## 3. Post-Processing of Blasting Vibration Data

### 3.1. Theoretical Analysis of the HHT Transformation Method

#### 3.1.1. EMD Decomposition

After vibration occurs, there are IMF components with non-sinusoidal characteristics, which constitute any complex signal, including the impact signal, and these IMF components are usually relatively simple [34,35]. These IMF components can be extracted through EMD, where EMD decomposition concludes with the residual component obtained in the final decomposition stage, as indicated by the algorithm expression.

(1) $$x\left(t\right)=\sum_{j=1}^{n}c_{j}\left(t\right){+r}_{n}\left(t\right),$$

In the equation, $c_{1}\left(t\right)$, c2(t), $c_{3}\left(t\right)$, …, cn(t) represent the components of the original signal in different frequency bands, collectively forming the original vibration signal, and their frequencies show a decreasing trend. The frequency bands of these small segments are not fixed, which proves the adaptive nature of EMD decomposition. rn(t) represents the calculated residual obtained in the final stage, and *n* denotes the number of iterations in the equation.

#### 3.1.2. Hilbert Transform

Performing the Hilbert transform on each decomposed IMF component yields the instantaneous frequency corresponding to each element, and the comprehensive integration of all immediate spectra results in the Hilbert spectrum [36]. The calculation steps are as follows:

Apply the Hilbert transform to all components, given by the following formula:

(2) $$H\left[c\left(t\right)\right]=\frac{1}{\pi }\cdot PV\cdot \int_{-\infty }^{\infty }\frac{c\left(t’\right)}{t-t’}dt’,$$

This formula represents the convolution of function c(t’) with function 1/πt.

In the equation, c(t’) represents a real-valued function, t’ is a real-valued function variable, and PV  is the Cauchy principal value. Based on this, an analytic signal z(t) is constructed.

(3) $$z\left(t\right)=c\left(t\right)+j\cdot H\left[c\left(t\right)\right]=a\left(t\right)\cdot e^{j\cdot \Phi \left(t\right)},$$

In the equation, j represents the analytic constant, a(t) represents the amplitude function, and Φ(t) represents the phase function. The formulas are as follows:

(4) $$\left\{ \begin{array}{l} a\left(t\right)=\sqrt{c^{2}\left(t\right)+H\left[c\left(t\right)\right]}\\ \Phi \left(t\right)=\arctan\frac{H\left[c\left(t\right)\right]}{c\left(t\right)} \end{array}\right.,$$

In the equation, c(t) represents the IMF component of the signal x(t).

Based on this, the instantaneous frequency ω(t) and instantaneous frequency f(t) are obtained, with the formulas as follows:

(5) $$\left\{ \begin{array}{l} \omega \left(t\right)=\frac{d\Phi \left(t\right)}{dt}\\ f\left(t\right)=\frac{1}{2\pi }\cdot \frac{d\Phi \left(t\right)}{dt} \end{array}\right.,$$

In the equation, Φ(t) represents the phase function.

After applying the Hilbert transform, the expression of the original signal is

(6) $$X\left(t\right)=R_{e}\cdot \sum_{i=1}^{\infty }a_{i}\left(t\right)\cdot e^{j\cdot w_{i}\left(t\right)},$$

In the equation, $R_{e}$ represents the real part of the residual component r, $a_{i}\left(t\right)$ and $w_{i}\left(t\right)$ are constants, and i is the index of the IMF component.

The representation of the amplitude displayed in the time–frequency domain is called the Hilbert spectrum, expressed as

(7) $$H\left(\omega ,t\right)=R_{e}\cdot \sum_{i=1}^{n}a_{i}\left(t\right)\cdot e^{\int w_{i}\left(t\right)dt},$$

In the equation, ω represents a specific frequency.

Integrating the Hilbert spectrum concerning time “*t*” yields the Hilbert marginal spectrum.

(8) $$h\left(\omega \right)=\int_{0}^{t}H\left(\omega ,t\right)dt,$$

The Hilbert instantaneous energy spectrum is an additional result derived from the Hilbert spectrum, and it can be defined as follows:

(9) $$IE\left(t\right)=\int_{0}^{t}H^{2}\left(\omega ,t\right)d\omega ,$$

### 3.2. Vibration Monitoring Results and Wavelet Soft Threshold Denoising

**Figure 4:**
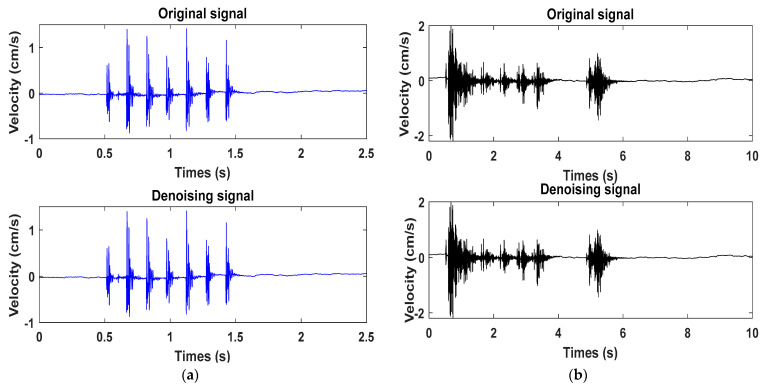
Original signal and denoised signal: (**a**) digital electronic detonator original signal and denoised signal; (**b**) the nonel detonator’s original signal and denoised signal.

The vibration signal measured by monitoring point 1, closest to the blast source, was analyzed. The seismic resistance of the building in the horizontal direction is relatively weak. When subjected to the same intensity flat load, the building is more prone to shear damage. During blasting, the vibration energy is transmitted to the upper part of the building through the foundation, and the height of the building amplifies the vibration response. Therefore, the horizontal tangential components of the two sets of blasting vibration signals at monitoring point 1 were analyzed. In addition, to eliminate the noise signal in the original signal and retain the valuable components of the original signal as much as possible, the wavelet soft threshold method was used to denoise the initial vibration wave. The “db 5” wavelet basis was used to decompose the original signal into five layers, and the wavelet decomposition coefficients were extracted. The denoising soft threshold was given, and the coefficients exceeding the empirically set threshold were processed accordingly to achieve the denoising purpose. Finally, the denoised signal was reconstructed through the inverse wavelet transform. Figure 4a,b show the vibration signals before and after denoising for the electronic and nonel detonators.

### 3.3. EMD Decomposition and Analysis

**Figure 5:**
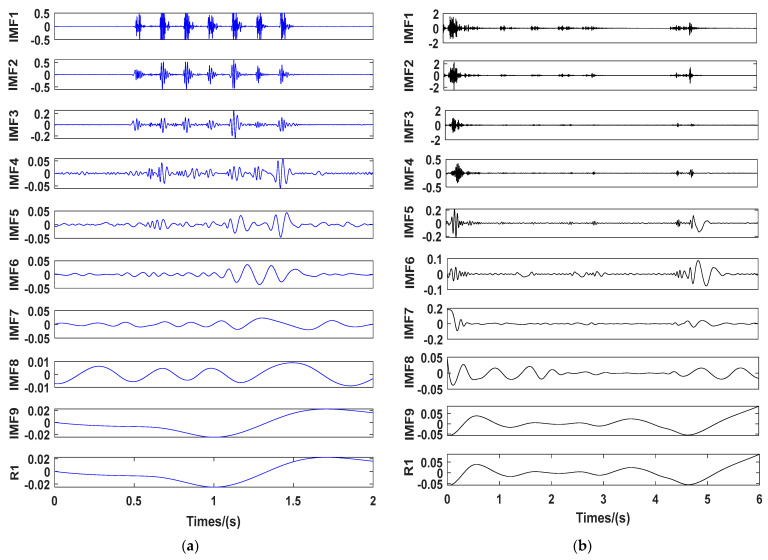
EMD decomposition results: (**a**) characteristic graphs of each IMF component of the digital electronic detonator; (**b**) characteristic graphs of each IMF component of the nonel detonator.

**Figure 6:**
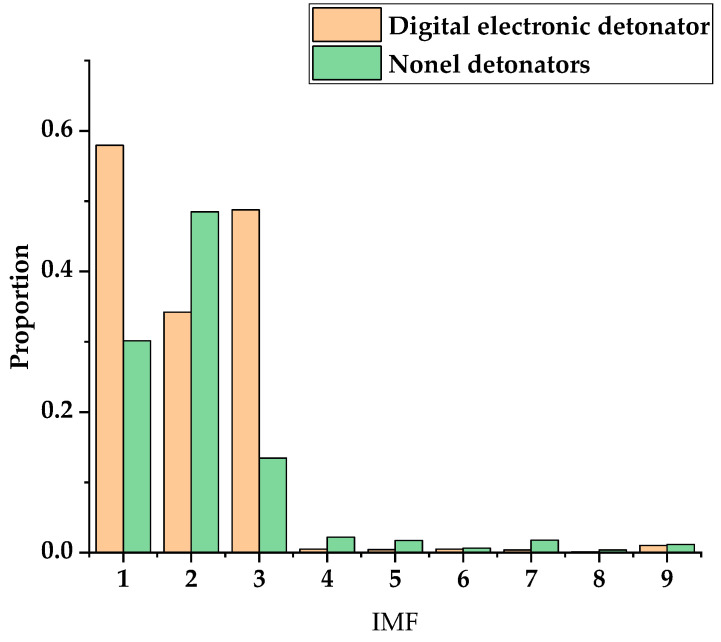
Bar graph showing the energy proportion of each IMF component for the digital electronic and nonel detonators.

Figure 5 and Figure 6 show the results of empirical mode decomposition (EMD) of the digital electronic detonator and nonel detonator. Figure 5a,b show the characteristic graphs of each IMF component after the EMD decomposition of the digital electronic and nonel detonator. Figure 6 shows the energy proportion of each digital electronic and nonel detonator’s IMF component. EMD was performed in 10 layers in sequence from high frequency to low frequency for the original blasting vibration signals of the two types of detonators. R1 represents the signal residue obtained after the final decomposition. As shown in Figure 5a and Figure 6, IMF 1-IMF 3 were the main components of the original signal. As the decomposition frequency gradually decreased, the waveforms gradually elongated, and the contained energy and amplitude decreased accordingly, slowly losing the characteristics of the original signal with the increase of decomposition layers. Finally, the residual component obtained after decomposition showed that the original signal was completely decomposed, and the residual part was generally ignored. The IMF 1 piece had the largest vibration amplitude and the highest energy for digital electronic detonators and can best represent the original signal. For the nonel detonators, IMF 1–IMF 3 contained the vast majority of the original signal’s energy and were the main components of the original signal. Among them, IMF 2 had the most energy in each IMF layer and was the main component of the original signal. Therefore, IMF 1 and IMF 2 were selected as the dominant components for the digital electronic detonator and the detonating cord detonator, respectively, for further decomposition analysis.

### 3.4. Identification of Actual Delay Time and Delay Error

Due to the delay error of the digital electronic detonators and nonel detonators used in field blasting tests, it is necessary to identify the actual delay time before comparing the time–frequency energy characteristics of the two detonators. Currently, many scholars use the instantaneous energy method to determine the actual delay time of the detonators, and the analysis object is the entire original signal. In contrast, the EMD delay identification method identifies a specific IMF component that can best represent the original signal, which reduces the interference in the original signal and allows for more accurate identification of the actual delay time of the detonator. As analyzed in the previous section, the IMF 1 component of the digital electronic detonator has the highest amplitude, contains the most energy, and has a waveform characteristic closest to the original signal. Therefore, IMF 1 component was selected to calculate its envelope spectrum for the electronic detonator. Similarly, the nonel detonator’s IMF 2 component was chosen to calculate the envelope spectrum for actual delay time identification.

**Figure 7:**
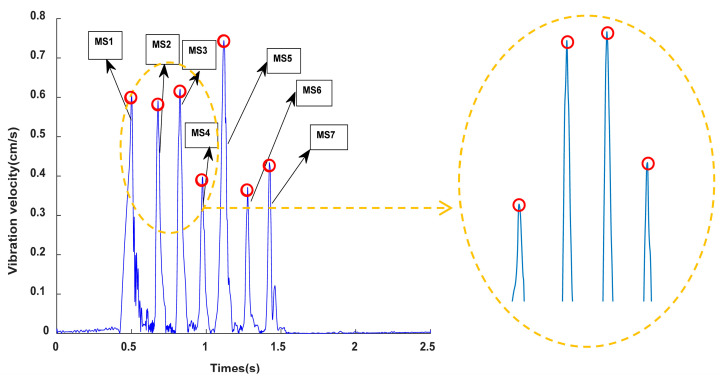
Envelope spectrum and detailed display of the IMF1 component signal of the digital electronic detonator.

**Figure 8:**
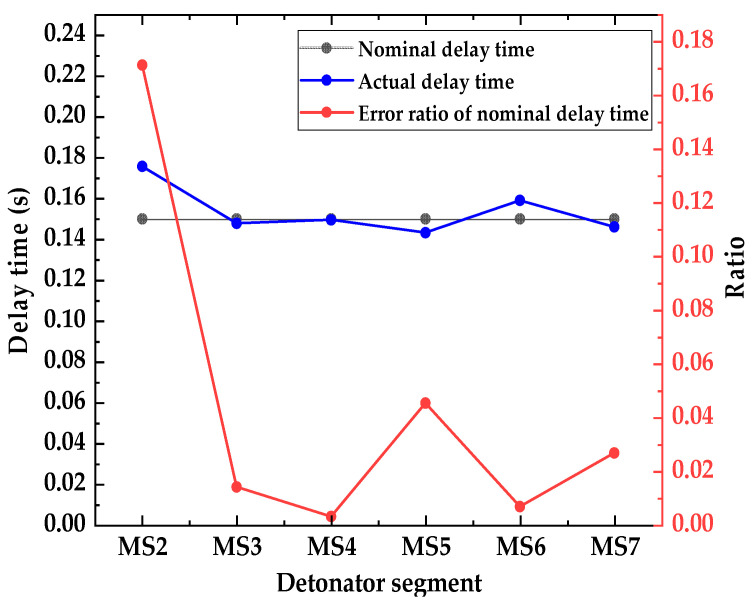
Relationship curve between nominal delay time, actual delay time, and error ratio of the digital electronic detonator.

Figure 7 shows the envelope spectrum and detailed display of the IMF 1 component signal of the digital electronic detonator obtained by EMD decomposition. Figure 8 shows the relationship curve between the nominal delay time, actual delay time, and the error ratio of the digital electronic detonator. The ratio of the actual delay error to the nominal delay time is defined here as the delay time error ratio. First, the actual delay time of the digital electronic detonator was identified. The envelope diagram in Figure 7 shows seven peaks of different amplitudes independent of each other. Each spike corresponds to one of the digital electronic detonators’ seven staged bursts. By examining the details of the envelope spectrum, it can be found that the digital electronic detonator can produce segmented waveforms with good integrity, and each segment has only one waveform peak, achieving maximum synchronous blasting within each piece. This indirectly proves the precise delay of digital electronic detonators. MATLAB signal analysis software was used to mark the data. The time corresponding to each peak in Figure 7 is displayed as 0.5018 s, 0.6775 s, 0.8253 s, 0.9748 s, 1.118 s, 1.277 s, and 1.423 s, with 6 delay time intervals corresponding to 7 segments of detonators being 0.1757 s, 0.1478 s, 0.1495 s, 0.1432 s, 0.159 s, and 0.146 s, respectively. Further calculations revealed that the delay time deviations between the actual and designed delay times for each detonator segment were 25.7 ms, 2.2 ms, 0.5 ms, 6.8 ms, 1 ms, and 4 ms, with an average delay time deviation of 6.7 ms and a delay accuracy of 95.7%.

In Section 2, we combined the parameters design of the blasting hole network with the testing process. it can be found that the first detonator segment is used for the slot excavation blasting and is the initiation area where there is only one free surface for blasting, and the rocks at the bottom of the blast hole are tightly packed, leading to a long duration of the shock wave inside the rock, which causes waveform movement. Therefore, the actual delay time of the first detonator segment has a poorer reference value for the overall blasting activity. After excluding the actual delay time data of the first segment, the actual delay times of the remaining 6 segments were distributed around the nominal delay time, with an average delay error of 2.9 ms and a delay accuracy of 98.1%, demonstrating a high recognition accuracy of the EMD delay identification method. The electronic detonators used in this blasting campaign met the accuracy requirement of having errors less than 1.5 ms within a 150 ms delay time, and the vibration measuring instrument met the national level A standard. Neither of these factors affect the analysis results. The average delay error of each segment identified by the EMD delay identification method in this blasting was 2.9 ms, but this does not mean that the electronic detonator used did not meet the accuracy requirements or that the recognition accuracy of the EMD delay identification method is problematic. Due to the simultaneous blasting of multiple blast holes within the segmented blasting, the delay time setting for each blast hole within the same segment was set at 150 ms, with a delay time error of ±1.5 ms for each electronic detonator in each blast hole. Therefore, it was not strictly simultaneous blasting.

**Figure 9:**
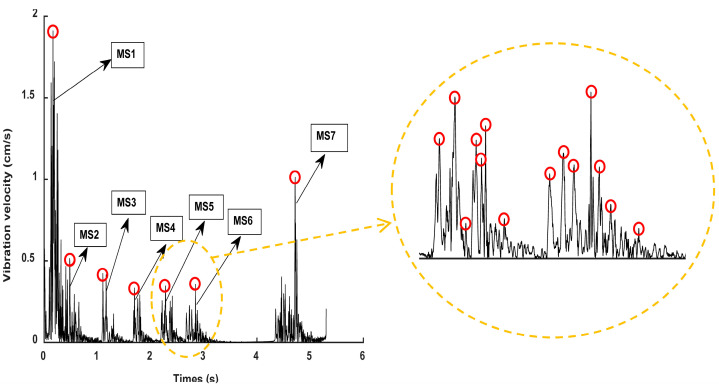
Envelope spectrum and detailed display of the IMF1 component signal of the nonel detonator.

**Figure 10:**
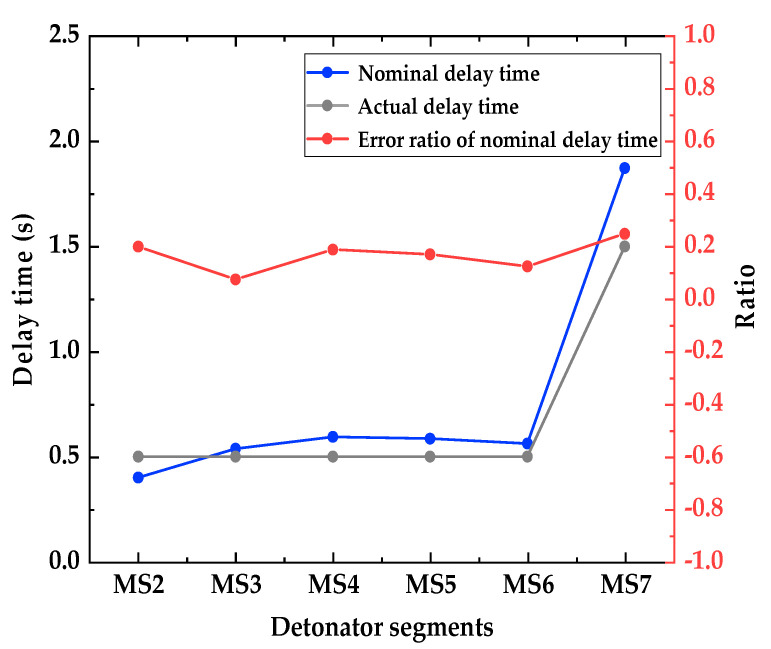
Relationship curve between nominal delay time, actual delay time, and error ratio of the nonel detonator.

Figure 9 shows the envelope spectrum and detailed display of the IMF 2 component signal of the nonel detonator. Figure 10 shows the relationship curve between the nominal delay time, actual delay time, and error ratio. The nonel detonator used in this blasting project has a delay time of half a second, with a permissible delay error of ±150 ms. Analysis of Figure 8 identifies seven segments of the blasting waveform, with only the first and second segments being superimposed, while the other five segments are relatively independent. The time of each waveform peak was 0.1665 s, 0.5667 s, 1.104 s, 1.698 s, 2.283 s, 2.845 s, and 4.718 s. Figure 10 shows that the actual delay times of the nonel detonators in the first six segments of the blasting conduit were generally distributed around the nominal delay time, with delay errors ranging from 0.0746 to 0.2478. In the seventh segment, the ninth nonel detonator was used. However, since this segment spans three parts and the delay interval is calibrated at 1.5 s with a delay error ratio of 0.2487, the nominal and actual delay time errors differed significantly and, therefore, could not be used as a reference for subsequent calculations. Thus, the maximum positive delay time error of the nonel detonator was +94 ms, the maximum negative delay time error was −99.8 ms, and the average error was 75.6 ms with an accuracy of approximately 84.9%. It can be seen that the use of nonel detonators did not achieve simultaneous blasting within each segment due to its delay accuracy issues.

Furthermore, by comparing Figure 7 and Figure 9, it can be observed that the envelope signals of the vibration waves of the digital electronic detonator blasting were relatively smooth. In contrast, the nonel detonator showed abrupt changes in the waveform within each segment, with many harmonics present. Figure 9 shows that in the detailed display of the 5th and 6th segments of the nonel detonator, at least seven waveform peaks with different amplitudes were found, with time intervals ranging from 14 ms to 43 ms, with an average interval time of 25.8 ms. In addition, the decay time of the vibration waves of each segment of the electronic and nonel detonator was identified and calculated, and the average decay times were 0.1089 s and 0.4484 s, respectively. The vibration wave decay rate of the digital electronic detonator was approximately four times that of the nonel detonator, with all parameters other than the detonator used being the same. Therefore, based on the above-shown analysis, it can be further confirmed that the digital electronic detonator has high delay accuracy and can essentially achieve simultaneous blasting within each segment. In contrast, the nonel detonator has more significant delay time errors, which lead to the vibration waves of each borehole within each segment being randomly superimposed based on the delay time control. This causes a slower vibration wave decay rate and more random peak values in the waveform within each segment.

## 4. Discussion

### 4.1. Comparative Analysis of Frequency Band Energy Distribution Characteristics

**Figure 11:**
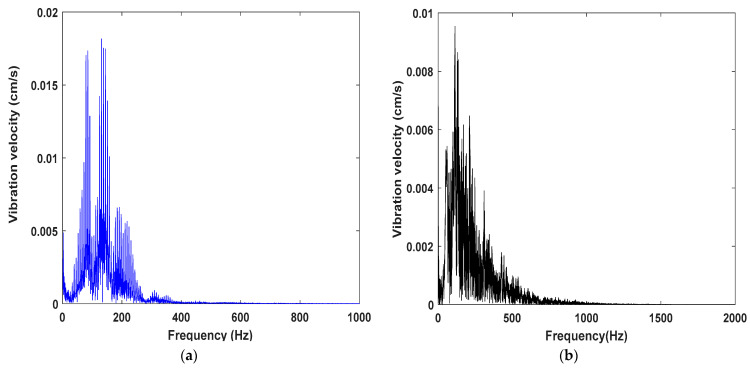
Blast vibration signal spectrum: (**a**) spectrum of the blasting vibration signal of the digital electronic detonator; (**b**) spectrum of the blasting vibration signal of the nonel detonator.

Figure 11a and Figure 11b, respectively, illustrate the distribution characteristics of vibration energy on vibration frequencies for digital electronic detonators and nonel detonators. By comparing Figure 11a and Figure 11b, it can be observed that the blasting energy of the digital electronic detonator was mainly concentrated within the frequency band range of 60–230 Hz, with a main vibration frequency band of 70–150 Hz and the primary vibration frequency of 86 Hz. In comparison, the blasting energy of the nonel detonator is mainly distributed within 600 Hz, with a main vibration frequency band of 80–200 Hz and a primary vibration frequency of 113 Hz.

It can be concluded that the main frequency energy of the digital electronic detonator blasting is higher than that of the nonel detonator, and the main frequency band of the electronic detonator is narrower, with most of the energy concentrated within the main vibration frequency band and a lower primary vibration frequency than the nonel detonator. By the second section of the identification of the delay time error, preliminary analysis indicates that under the same working conditions, the delay time error of nonel detonators is highly likely to cause the vibration waves from each single-hole blast in the same section to overlap with each other and generate numerous harmonic frequencies, resulting in a broadening of the main vibration band. Additionally, the envelope spectrum shows that the main vibration frequency and harmonic frequencies of nonel detonators exhibit a disorderly distribution. This ultimately leads to the distribution of blasting energy on a broader frequency band, avoiding the concentration of energy in the main vibration frequency. The superposition effect of the vibration wave caused by the delay error reduces the energy of the primary frequency. However, since low-frequency energy is the leading cause of inducing resonance damage to buildings and structures, it cannot be concluded that nonel detonators protect the buildings and structures under resonance conditions. A comparison of the distribution of low-frequency energy between digital electronic detonators and nonel detonators is necessary.

**Figure 12:**
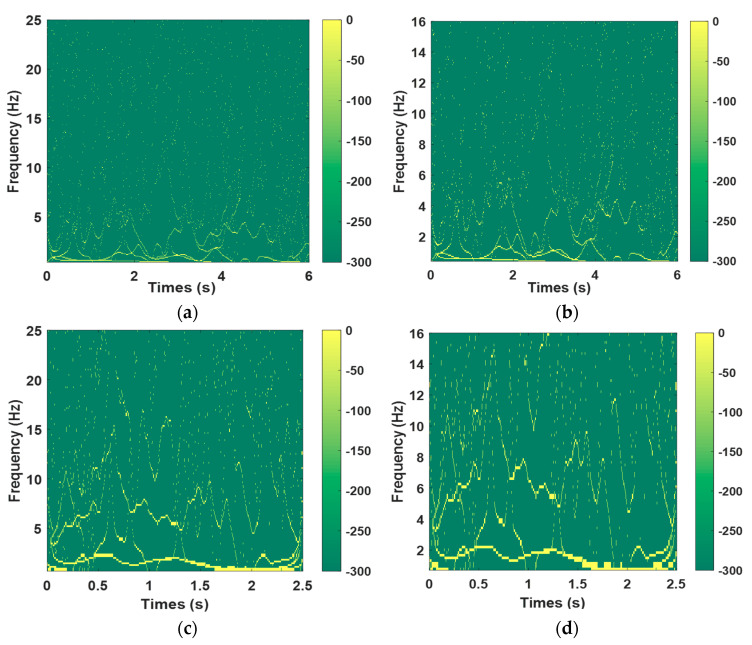
2D Hilbert spectra of vibration signals from digital electronic detonators and blasting conduit detonators: (**a**) 2D Hilbert energy spectrum of the digital electronic detonator within the frequency range of 0–25 Hz, (**b**) 2D Hilbert energy spectrum of the digital electronic detonator within the frequency range of 0–16 Hz, (**c**) 2D Hilbert energy spectrum of the nonel detonator within the frequency range of 0–25 Hz, and (**d**) 2D Hilbert energy spectrum of the nonel detonator within the frequency range of 0–16 Hz.

**Figure 13:**
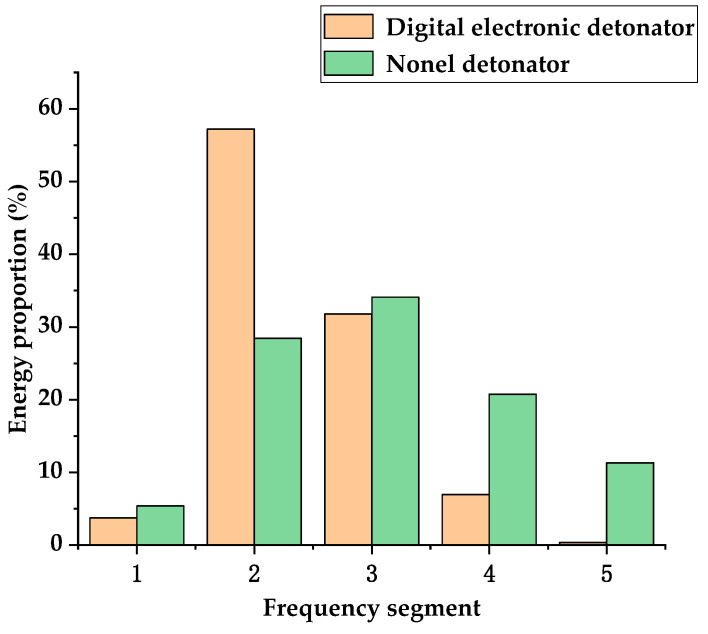
The energy proportions within each frequency band during blasting using digital electronic detonators and nonel detonators.

Figure 12a and Figure 12b, respectively, show the 2D Hilbert energy spectrum of the digital electronic detonator within the frequency range of 0–16 Hz and 0–25 Hz. Figure 12c,d shows the 2D Hilbert energy spectrum of the nonel detonator within the frequency range of 0–16 Hz and 0–25 Hz. Figure 13 shows the energy proportion of the digital electronic detonator and the nonel detonator in the frequency bands of 0–50 Hz, 50–100 Hz, 100–200 Hz, 200–300 Hz, and over 400 Hz. Specific details are provided in Table 1. Observing Figure 12, it can be found that both the digital electronic detonator and the nonel detonator have energy distribution within the low-frequency bands of 0–25 Hz, especially within 0–10 Hz, as shown by the color code on the right side of Figure 11, where the closer to yellow represents a higher concentration of energy, and the dense yellow spots in the figure indicate where the energy is concentrated. Specifically, when using digital electronic detonators for blasting, the low-frequency energy is mainly distributed within the frequency band of less than 5 Hz.

In contrast, using nonel detonators in blasting generates more low-frequency energy. To more intuitively display the vibrational energy distribution within each frequency band, the frequency range was further divided into 0–50 Hz, 50–100 Hz, 100–200 Hz, 200–300 Hz, and greater than 300 Hz frequency bands. The energy proportion of each frequency can be obtained by integrating the frequency within each of the five frequency bands, as shown in Figure 13. The energy proportion of the digital electronic detonator and the nonel detonator within five frequency bands is shown in Table 1. As shown in Table 1, for the low-frequency energy of 0–50 Hz, the proportion of nonel detonators was 5.39%, higher than the 3.73% digital electronic detonator. However, the contribution to the total energy was relatively small, and the possibility of low-frequency energy-inducing building resonance was low. Therefore, it does not conflict with the conclusion that the nonel detonator provides more excellent protection to structures under resonance conditions than the digital electronic detonator. When using digital electronic detonators for blasting, the energy distribution was concentrated within the frequency band of 50–200 Hz, with an energy proportion of up to 88.98%. In contrast, when using the nonel detonator for blasting, the energy was relatively evenly distributed within the frequency bands of 50–100 Hz, 100–200 Hz, 200–300 Hz, and greater than 300 Hz. The energy proportions within the frequency bands were 28.44%, 34.1%, 20.76%, and 11.32%, respectively. The characteristics of the nonel detonator, which distributes vibrational energy more evenly across frequency bands and concentrates less energy at the primary vibration frequency, suggest that nonel detonators provide more excellent protection to structures compared to digital electronic detonators under the working condition of simultaneous blasting in small cross-section roadway sections.

### 4.2. Chaotic Superposition of Vibration Waves Induced by Delay Errors for Vibration Reduction

**Figure 14:**
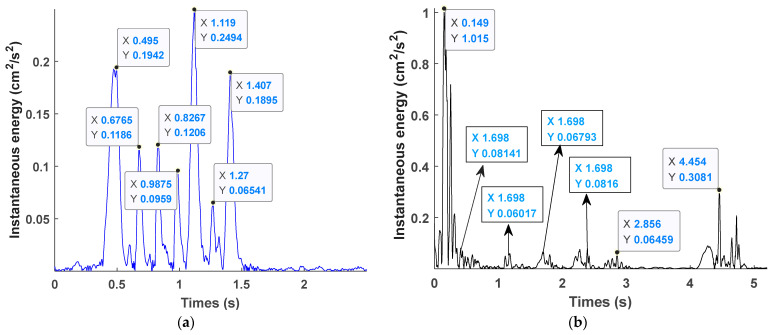
Instantaneous energy graph: (**a**) digital electronic detonator; (**b**) nonel detonators.

Figure 14a and Figure 14b, respectively, present the instantaneous energy spectra of vibration signals of digital electronic and nonel detonators, which can reflect the characteristics of energy changes over time. It can be observed that the maximum instantaneous energy of the digital electronic detonator appeared at 1.119 s, corresponding to the detonation of the 5th segment detonator, which includes the bottom eye, assistant eye, and the first circle of pressure eye. As indicated in the blasting design shown in Figure 1, this section consumed 54 rolls of emulsified explosives. Although the previous sections generated a blast-free surface, the number of explosives destroyed in this section was much higher than in the other six sections, generating more energy. The maximum instantaneous energy of the nonel detonator appeared in the first segment detonator blast, which is the slot blasting. Generally, detonation under a single free surface condition with relatively weak external effects is subjected to an enhanced rock clamp effect, leading to a relatively high proportion of vibration energy converted from explosive power. However, as analyzed above, the main reason is that the vibration waveforms of the first and second sections of the nonel detonator were superimposed. For the fifth segment, which consumed the highest amount of explosives, the blasting vibration energy generated by nonel detonators was much lower than that generated by digital electronic detonators. Preliminary analysis results indicate that the detonator’s only variable, delay time errors, induced chaotic superposition of vibration waveforms within each borehole, which significantly reduced vibration.

Further statistical analysis was conducted on the vibration energy generated by the two types of detonators: nonel and electronic. Since the delay time error of the first segment of the nonel detonator is zero, there is no delay error. Additionally, the vibration waves generated by the first and second segments of the nonel detonator overlapped. Therefore, the energy changes of the first and second segments were not used as reference data when discussing the possibility of vibration wave disorderly superposition and vibration reduction caused by the delay time error between the holes of the nonel detonator. Further statistical analysis was conducted on the blasting energy of segments 3–7. The average instantaneous energy of the electronic and nonel detonators was 0.144 cm^2^/s^2^ and 0.116 cm^2^/s^2^, respectively. The average vibration reduction rate of each segment of the nonel detonator compared to the electronic detonator was 19.4%. Further discussion was conducted on the possibility of vibration wave disorderly superposition and vibration reduction caused by the delay error of the nonel detonator.

Based on the periodicity of blasting vibration waves, although their waveforms are not entirely consistent with sine waves, the interference and superposition between blasting vibration waves can still be explored by referring to the superposition rule of sine waves [37]. When sine waves propagate in the same medium with the same period, there is T=2π/ω, and the phase angle of the two sine waves is determined by $0\leq \varphi_{1}\leq \varphi_{2}\leq 2\pi /\omega$.

Two sine waves are represented as $A_{1}$ and $A_{2}$. Thus, we have

(10) $$A_{1}=\sin(\omega t-\varphi_{1}),$$

(11) $$A_{2}=\sin(\omega t-\varphi_{2}),$$

To superimpose the two sine waves, the formula is expressed as

(12) $$A=2\sin(\omega t-\frac{\varphi_{1}+\varphi_{2}}{2})\cos(\frac{\varphi_{2}-\varphi_{1}}{2}),$$

Based on the characteristics of sine waves, in the formula $-1\leq A_{1}\leq 1$,$-1\leq A_{2}\leq 1$ and t∈(0,∞), plug in the following procedure:

(13) $$-1<\sin(\omega t-\frac{\varphi_{1}+\varphi_{2}}{2})<1,$$

It is necessary to satisfy this condition to achieve the purpose of superimposing and weakening two sine waves −1≤A≤1. According to Formulas (12) and (13), we have

(14) $$-\frac{1}{2}<\cos(\frac{\varphi_{2}-\varphi_{1}}{2})<\frac{1}{2},$$

Furthermore, $\varphi_{2}-\varphi_{1}$ it must satisfy the following equation:

(15) $$2\pi /(3\omega )<\varphi_{2}-\varphi_{1}<4\pi /(3\omega ),$$

When the phase angle satisfies the condition of Equation (15), it can be considered that the two waves are superimposed and weakened. According to the definition of a sine wave, the phase difference represented by Equation (15) can also be regarded as the time interval between the two waves when they start to propagate. The angular velocity of the wave with a period of *T* can be expressed as ω=2π/T. Finally, substituting into the above-shown equation:

(16) $$kT+T/3<\Delta t_{1}<kT+2T/3,$$

The $\Delta t_{1}$ represents the time interval between the propagation of the two waves and refers to the delay interval in this context. When the delay interval satisfies Equation (16), the two waves produce different degrees of superimposition and weakening.

**Figure 15:**
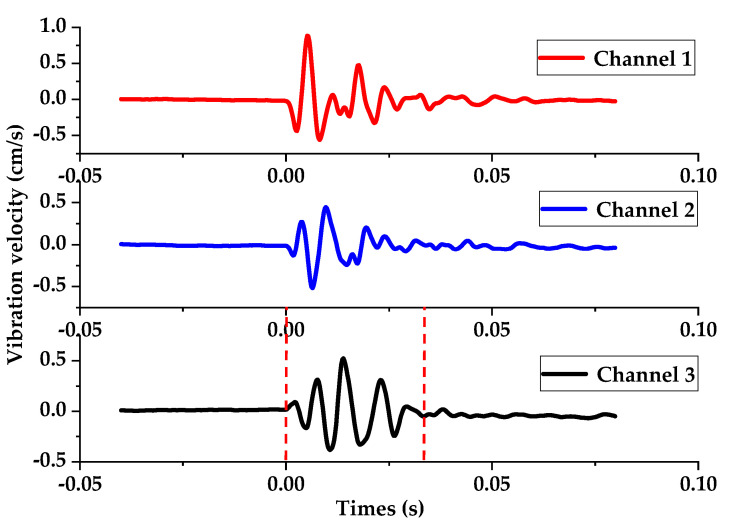
Vibration waveform of three channels for single-hole blasting initiated by the nonel detonator.

In addition to vibration testing during the excavation blasting of the tunnel, a single-hole blasting test was also designed with all parameters consistent with those of the collapsed hole blasting parameters when the nonel detonator was used. The measured vibration waveform is shown in Figure 15, and it was found through MATLAB software’s data cursor function that the attenuation time of the vibration waveform of the single-hole blasting was 40 ms. The two red dashed lines in the figure represent the start and end positions of the vibration waveform for this single-hole blasting. Therefore, T=40 ms was substituted into Equation (16). When the delay error $\Delta t_{1}$ of each detonator meets K×40+10<Δt<K×40+20, the single-hole waveforms within the section can interfere with each other and weaken each other’s vibration, achieving the purpose of interference reduction. Further calculations showed that when the error delay of the nonel detonator was within the delay interval $\Delta t_{1}$ of 10–20 ms, 50–60 ms, 90–100 ms, and 130–140 ms, the phenomenon of interference reduction of the single-hole vibration waveform would occur. In other delay intervals within 0–150 ms, vibration waves were instead superimposed, enhancing the vibration effect.

Thus, under the control of delay time error, both superimposed attenuation and enhancement of vibration waves would occur. However, the phenomenon of enhancement or attenuation is relative to the vibration of a single blast hole. Therefore, regardless of whether the single-hole blasting vibration waveform is superimposed or attenuated, its peak value will be reduced compared to the simultaneous blasting vibration. Based on the randomness of the delay time error, we cannot investigate the specific superposition situation of the vibration waveform under the control of delay time error. However, the comparison of vibration energy shows that nonel detonators undergo unordered superposition and interference reduction under the supervision of delay error. When blasting simultaneously between sections, the delay error of the nonel detonator plays a positive role in vibration control.

### 4.3. Comparison of Blasting Effects

**Figure 16:**
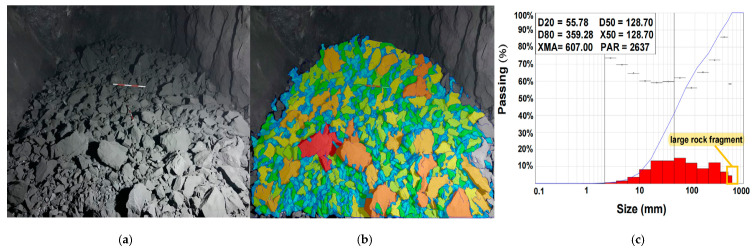
Digital electronic detonator blasting rock size analysis: (**a**) on-site shooting of blasting pile samples; (**b**) sample processing diagram; (**c**) WipFrag analysis chart.

**Figure 17:**
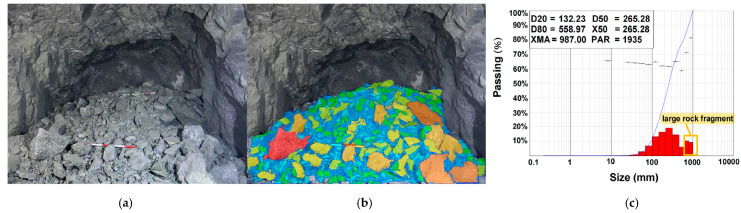
Nonel detonator blasting rock size analysis: (**a**) on-site shooting of blasting pile samples; (**b**) sample processing diagram; (**c**) WipFrag analysis chart.

The previous section mainly conducted a comparative analysis of digital electronic and nonel detonators under the same working conditions from the perspective of time-frequency-energy analysis. It is difficult to evaluate the two types of detonators solely from the standpoint of vibration propagation. Therefore, it is necessary to compare and analyze the blasting effects of the two kinds of detonators. Figure 16 and Figure 17 present the results of particle size analysis for blasting. Firstly, the sample photos of blasted rock piles were taken on-site. Then the WipFrag software was used to analyze the digital electronic and nonel detonator blasting particle size. The degree of rock fragmentation was evaluated by the cumulative content of broken rocks at 20% (D20), 50% (D50 or X50), and 80% (D80), the proportion of large broken rocks, the rate of over-breakage, and the maximum rock size. D20, D50/X50, and D80 represent the rock fragmentation sizes at which the cumulative content reaches the corresponding percentage. The analysis results show that the D50/X50 values of the digital electronic and nonel detonator blasting were 128.7 mm and 265.28 mm, respectively. The values of D20 were 55.78 mm and 132.23 mm, respectively. The importances of D80 were 359.28 mm and 558.97 mm, respectively. These three statistical values indicate that the particle size of the rock fragmentation by the digital electronic detonator is smaller than that by the nonel detonator, which is more in line with the excavation requirements.

According to the requirements of the iron ore beneficiation plant, particles with a diameter above 600 mm are defined as large-sized particles. In comparison, particles with a diameter below 25 mm are described as small-sized particles. Data analysis of the histograms shows that using digital electronic and nonel detonators resulted in a significant size rate of 19.66% and 4.6% and an over comminution rate of 0.18% and 2.37%, respectively. The maximum size of broken rocks is 607 mm and 987 mm, respectively. Furthermore, the histogram demonstrated a more uniform distribution of rock fragmentation sizes when using digital electronic detonators. Therefore, overall, using digital electronic detonators results in a lower rate of large-sized rock fragments and smaller size of broken rocks, which is more suitable for loading and transportation requirements.

## 5. Conclusions

By conducting on-site experiments and analyzing vibration waves, we comprehensively compared the differences between digital electronic and nonel detonators in small-section rock tunnel construction and arrived at the following main conclusions.

The vibration attenuation speed of digital electronic detonators is four times that of nonel detonators. Still, nonel detonators have higher energy at the primary frequency and a narrower main vibration frequency band, resulting in a broader distribution of blasting energy.

When the delay error of nonel detonators was randomly distributed within specific intervals, interference-damping phenomena occur. Nonel detonators exhibited an average damping rate of 19.4% compared to digital electronic detonators.

The delay error in the nonel detonators section, where blast holes are initiated, plays a positive role in vibration control. Additionally, it has lower costs and still meets the requirements for blasting fragmentation, albeit slightly inferior. This provides a new reference for future mixed initiation using digital electronic detonators and nonel detonators.

## Figures and Tables

**Table 1:** The energy proportion of digital electronic and nonel detonators in each frequency band.

Frequency Range	Energy Proportion in Each Frequency Band as a Percentage/%				
	0~50 Hz	50~100 Hz	100~200 Hz	200~300 Hz	>300 Hz
Electronic detonator	3.73	57.2	31.78	6.94	0.36
Nonel detonator	5.39	28.44	34.1	20.76	11.32

## Data Availability

Not applicable.
